# Effect of dehydroepiandrosterone on meiotic spindle structure and oocyte quality in mice

**DOI:** 10.22038/IJBMS.2018.27111.6629

**Published:** 2018-10

**Authors:** Ali Reza Eftekhari Moghadam, Ghasem Saki, Masoud Hemadi, Zohre Mazaheri, Ali Khodadadi

**Affiliations:** 1 Physiology Research Center, Faculty of Medicine, Ahvaz Jundishapur University of Medical Science, Ahvaz, Iran; 2 Fertility and Infertility Research Center, Faculty of Medicine, Ahvaz Jundishapur University of Medical Sciences, Ahvaz, Iran; 3 Department of Anatomical Science, Faculty of Medicine, Tarbiat Modarres University, Tehran, Iran; 4 Department of Immunology, Faculty of Medicine, Ahvaz Jundishapur University of Medical Science, Ahvaz, Iran

**Keywords:** Dehydroepiandrosterone Meiotic spindle, Mice, Oocyte quality, Pregnancy

## Abstract

**Objective(s):**

Dehydroepiandrosterone (DHEA) has been reported to improve pregnancy chances in women with diminished ovarian reserve (DOR) and to reduce miscarriage rates by 50–80%. This study, therefore, assesses effects of DHEA on number of retrieved oocytes and meiotic spindles.

**Materials and Methods:**

A randomized, prospective, controlled study was conducted on eight groups, four groups of young mice and four elderly. All young and old groups received different oral doses (35, 50, 75 mg/kg) of DHEA for 3 months. Meiotic spindle assessment was done by immunocytochemical techniques using a confocal laser microscope (Leica TCS-4D).

**Results:**

Statistical surveys showed that in control young groups 80% (*P*=0.0845) and in the old control group 73.3% (*P*=0.000) of the meiotic spindles have a normal shape and structure; the difference was meaningful. The young with 50 mg/kg of DHEA in 85.4% and the young with 75 mg/kg of DHEA in 84.2% were normal in shape and structure. Statistical analysis showed that the difference was meaningless (*P*=0.845). The old group with 30 mg/kg of DHEA in 81.1%, the old with 50 mg/kg of DHEA in 83.9%, and the old with 75 mg/kg of DHEA in 79.0% showed normal shape and structure. The meiotic spindle disruption ratio in old mice showed a significant difference (*P*=0.000) in comparison with others in young groups. Statistical analysis showed that difference between DHEA and control groups is meaningful. But this difference was meaningless between DHEA groups.

**Conclusion:**

Results showed that DHEA has a positive and improvement effect on the meiotic spindle in old mice.

## Introduction

Studies show that chromosomal abnormalities are the major cause of abortion in the first trimester (1). It is estimated that 15-20% of recognized clinical gestation abortions are related to it (2, 3). Other causes of miscarriage are anatomical variations in the uterus, like bighorn uterus, immunological changes, environmental, etc (3).

Bettio *et al.* described chromosomal changes in aborted embryos that were fertilized with ART (Assisted Reproductive Technology) by PGD (Pre-Implantation Genetic Diagnosis) techniques (4). These findings led researchers to follow the main cause of miscarriage and find a solution to help them decrease the chromosomal related aneuploidy and consequently increase the pregnancy chance in ART (5).

It has been demonstrated that Dehydroepiandrosterone (DHEA) could improve embryo quality and the chance of pregnancy in women that underwent an ART (6, 7). Casson *et al.* were the first to describe the effect of DHEA on ovarian stimulation in poor responder women (8). DHEA is an important prohormone in ovarian follicular steroidogenesis (8, 9). It is an endogenous hormone that is mostly produced in the adrenal gland cortex (zona reticularis) and ovarian theca cells (9). In the adrenal gland DHEA is synthesized from cholesterol and converted to pregnenolone, then it is converted to estrogen and testosterone in target organs. The maximum secretion of this hormone is at age 21 and its amount is reduced by 90% at age 75, infact, DHEA has a comedown process with aging (9, 10). DHEA is highest in the early morning, but its serum content rapidly drops during the day by kidneys (11). It is known that this hormone could have a positive effect on oocyte and zygote qualities (12).

Studies show that chromosomal and spindle abnormalities become much more prevalent in oocytes with age and are considered major factors responsible for increasedincidence of infertility, fetal loss (miscarriage), and conceptions resulting in birth defects most notably in down syndrome in women over 35 years old (13). We know, indeed, that the microtubules of oocytes are vulnerable to environmental and aging-related changes like ROS, TNF, and other oxidative stresses (13). Gleicher *et al.* could decrease miscarriage rate in women who underwent ART by administration of 75 mg/kg/day of DHEA for 12 months and consequently, they observed a reduction in embryo aneuploidy by PGS techniques (14, 15).

Also in humans, it was shown, depolymerization of microtubules is sometimes attendant with disbanding of chromosomes and changes in oocyte qualities (13, 16). Although the microtubular system of mice with respect to the distribution of pericentriolar material is different from that of human oocyte, it has been routinely used as a model to study the spindle structure of human oocytes (17). Morphological oocyte evaluation, however, is still the standard criterion for routine work in ART (18). Morphological assessment before ART helps to identify metaphase II(MII) oocytes with higher developmental potential (19). The morphology of MII oocytes includes oocyte shape, color, granularity and homogeneity of cytoplasm, width of perivitelline space (PVS), debris in PVS,vacuolization, inclusions, and abnormalities of the first polar body (1^st ^PB) or of ZP (20). Our study, for these reasons, was performed by administration of DHEA and assessed the potential benefit of DHEA for the meiotic spindle shape and oocyte quality of two independent old (12–14 months old) and young (6 weeks old) female mice.

## Materials and Methods

***Chemical reagents***

All chemical reagents were purchased from the Sigma Chemical Co, St Louis, MO, USA, except for antibodies, which were purchased from Abcam Inc., Cambridge, MA, USA.

***Animal design study***

This study was conducted from July 2013 to September 2013.  A total of 40 adult female NMRI mice (weight 45±5.1 g) were purchased from Laboratory Animals Care and Breeding Centre of Ahvaz Jundishapour University of Medical Sciences, Ahvaz, Iran. All efforts were made to minimize the number of animals used and their suffering. The fertility of female mice was proven at the beginning of the experiment by selecting post-first-wave of folliculogenesis that mated and observing positive pregnancy (21). All mice were randomly divided into two equal young and old (12-14 months old female mice) groups (G1–G8) (n =5): control and experimental groups. All animals were housed individually per cage under a 12-hr light/dark cycle, at 20±2 °C in a 60-65% humidity-controlled room with food and water *ad libitum*. All procedures were approved by international guidelines and by the Institute of Research Ethics and Animal Care and Use Committee of Ahvaz Jundishapour University of Medical Sciences (P/8/20/D//4856). The experimental groups received different doses (35, 50, 75 mg/kg) of DHEA (a fine crystalline powder, Sigma-Aldrich Chemie GmbH) once a day for 12 weeks by gavage (22). G1: young group (control group) with no administration of DHEA. G2: young group received 35 mg/kg. G3: young group received 50 mg/kg.G4: received 75 mg/kg. G5: old group no administration of DHEA. G6: old group received 35 mg/kg. G7: groupreceived 50 mg/kg.G8: oldgroup received 75 mg/kg.

**Figure 1:**
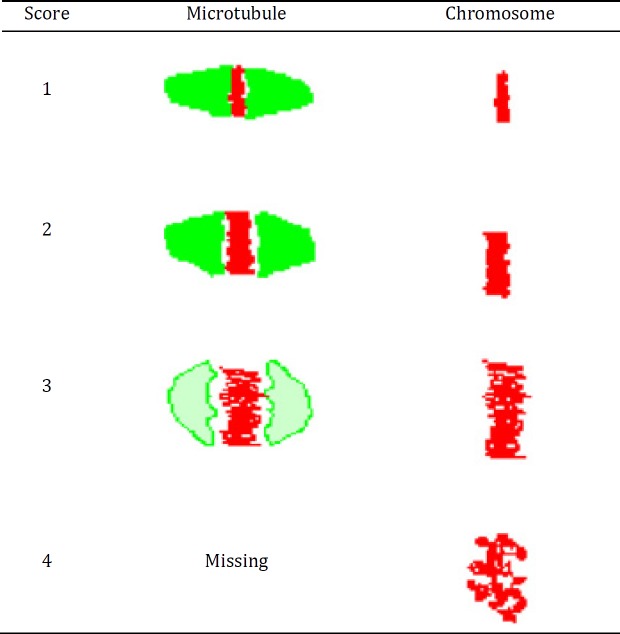
Diagrammatic representation of scoring criteria of microtubule and chromosomal changes based on microscopic evaluation (16)

**Figure 2:**
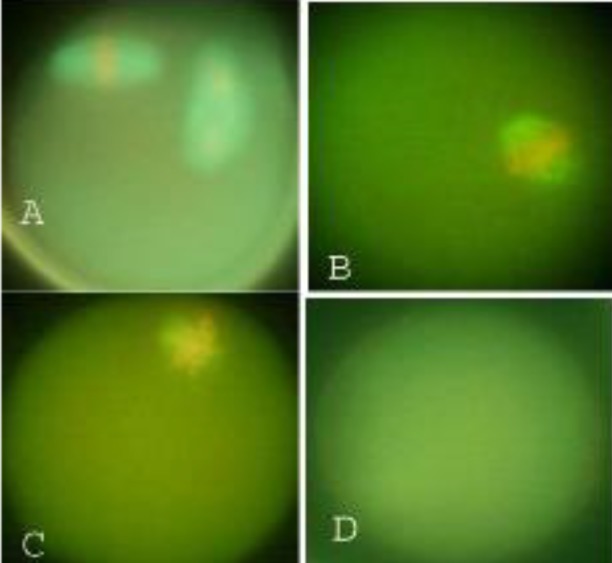
The configuration of meiotic spindle and chromosomes in studied groups. (A) Normal structure of spindles was barrel shaped with microtubules traversing between both poles and chromosomes typically (metaphase chromosomes) aligned regularly in a compact group on the equatorial plane oriented (B) Spindlemorphology included reduced size of the spindle,disrupted microtubule, completeabsence of a spindle, perpendicularly to the spindle axis

**Figure 3:**
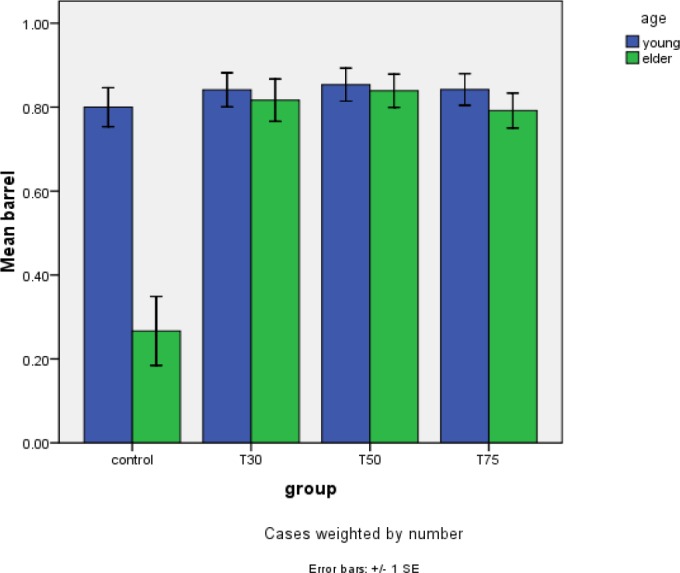
Barrel shaped structure in the study groups after DHEA

**Figure 4:**
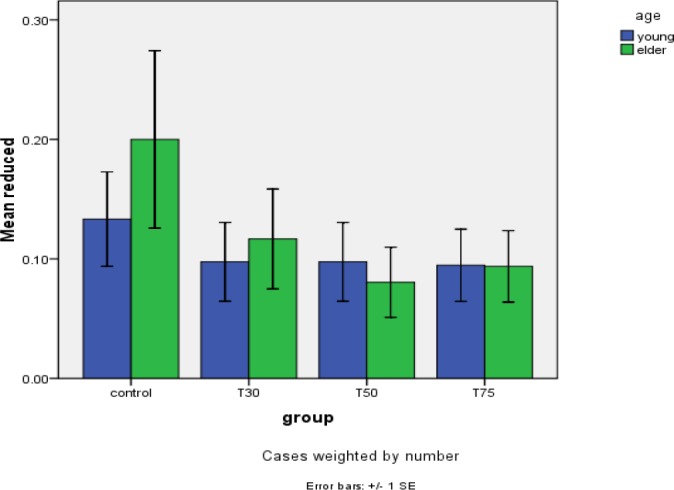
Reduced meiotic spindle morphology in study groups after DHEA

**Figure 5:**
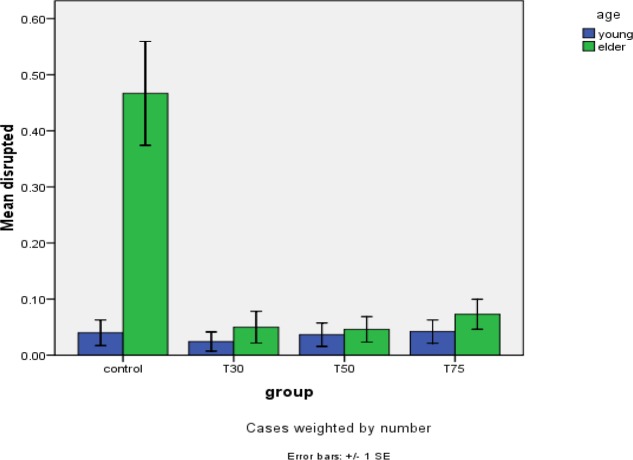
Meiotic spindle disruption in study groups after DHEA

**Figure 6:**
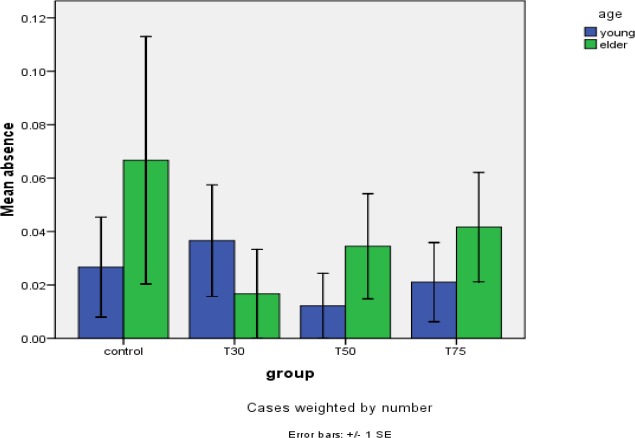
Absence of meiotic spindle in study groups after DHEA

**Table 1:** Number of retrieved oocytes in the study groups

	1ST MOUSE	2 TH MOUSE	3 TH MOUSE	4TH MOUSE	5 TH MOUSE	AVERAGE
G1	13	15	14	17	16	15±1.6
G2	18	16	20	10	17	16.2±3.8
G3	17	14	17	11	21	16±3.7
G4	20	16	19	16	24	19.2±3.3
G5	15	4	4	0	7	6±5.5
G6	17	7	13	8	15	12±4.6
G7	22	16	18	12	19	17.4±3.7
G8	21	18	16	19	22	19.2±2.4

***Oocyte collection and preparation***

Female NMRI mice received 5 international units of a pregnant mare’s serum gonadotropin (PMSG) injected intraperitoneally to induce superovulation. This was followed 46-48 hr later by the intraperitoneal administration of 5 IU human chorionic gonadotropin (HCG) (23). Twelve to 14 hr post-HCG injection mice were killed by cervical dislocation and oocytes were collected from the ampulla of the uterine tube under a loop microscope (Olympus, Japan). Oocytes were denuded of cumulus cells by using hyaluronidase washed with human tubal fluid (HTF) supplemented with BSA and classified under an inverted microscope, as MII (first polar body in perivitelline space), maturation arrested (germinal vesicle breakdown with no polar body extrusion, germinal vesicle intact), or degenerated (23).

***Spindle and chromosome staining***

Superovulated oocytes were denuded from cumulus cells, briefly incubated in acidified Tyrode’s solution to soften the zona pellucida, Fixation and all subsequent incubations were carried out at 37^ °^C. For microtubule staining, oocytes were fixed in a fixative solution (4% formaldehyde , 0.2% Triton X-100 in 100 ml PBS, goat serum) for 30 min and then incubated in anti α-tubulin monoclonal antibody (Abcam) (1:100) overnight at 4 ^°^C, followed by incubation in fluorescein isothiocyanate (FITC)-labeled anti-mouse IgG antibody (Abcam) (1:50) for 2 hr. For chromosome staining, oocytes were incubated in propidium iodide (1) (Sigma-Aldrich) (10 mg/ml) for 5 min (24). Each staining step was followed by a rinse in PBS for 5 min.

***Confocal microscopic analysis and scoring***

Slides were examined using a laser-scanning confocal microscope (Leica TCS-4D), equipped with an argon ion laser (excitation 488 nm, barrier 500–555 nm for FITC; excitation 568 nm, barrier 575–675 nm for PI). Microtubule distribution and chromosome alignment of each oocyte were examined. Scoring of morphological changes in microtubules and adjustment in the chromosomal alignment was scored according to a method described in the literature (24) with a slight modification (Figure 1). Briefly, spindle formation was categorized as good (scores 1, 2) when a condensed and barrel-shaped structure with slightly pointed poles formed by organized microtubules transverse from one pole to another was observed, and as abnormal or poor (scores 3, 4) when there was a reduction in the longitudinal dimension of the spindle or when there was partial or total disorganization; complete absence or remnant of intersperse and diffuse spindle was scored as 4. Chromosomes were regarded as good (scores 1, 2) when chromosomes were arranged in a compact metaphase plate at the equator of the spindle. The chromosomal organization was regarded as abnormal or poor (scores 3, 4) based on the degree of chromosomal displacement from the plane of the metaphase plate. When chromosomes were dispersed or showed less condensed appearance, score of 4 was divided (25).

## Results

Total oocytes were obtained from 40 adult mice 30 of which were treated with DHEA as shown in Table 1. The MII meiotic spindle assessments were done using a confocal laser microscope, and oocyte morphologic evaluation was carried out using a camera-equipped inverted microscope.

***Meiotic spindle structure assessment***

Confocal analysis of α-tubulin and chromosomal distribution revealed normal spindle structure was barrel-shaped with microtubules traversing between both poles, and chromosomes, typically the metaphase chromosomes, aligned regularly in a compact group on the equatorial pole, oriented perpendicularly to the spindle axis (Figure 2A). Abnormal spindle morphology included a decrease in the number of microtubules and the size of the spindle (Figure 2B), interruption of the spindle (Figure 2C), or complete absence of a spindle (Figure 2D)(16, 24). Table 1 shows the frequency of barrel-shaped spindles among treatment groups separately for young and old mice. In group 1, of 75 retrieved oocytes, 80% were barrel-shaped, 13.3% showed decreased size of meiotic spindle, 4% showed disruption in the meiotic spindle, and in 2.7% meiotic spindle was absent (Figure 3). In group 2, of 81 retrieved oocytes, 84% were barrel-shaped, 9.8% showed decreasedsize of meiotic spindle, 2.4% showed disruption in the meiotic spindle, and in 3.7% meiotic spindle was absent. In group 3, of 80 retrieved oocytes, 85.4% were barrel-shaped, 9.8% showed decreased size of meiotic spindle, 3.7% showed disruption in the meiotic spindle, and in 1.2% meiotic spindle was absent. In group 4, of 95 retrieved oocytes, 84.2% were barrel-shaped, 9.5% showed decreased size of meiotic spindle, 4.2% showed disruption in the meiotic spindle, and in 2.1% meiotic spindle was absent (Figure 4). Statistical analysis showed that different doses of DHEA do not have any effect on the shape of the meiotic spindle (*P*<0.005). In group 5 (old control), of 30 retrieved oocytes, 26.7% were barrel-shaped, 20% showed decreased meiotic spindle size, 46.7% showed disruption in the meiotic spindle, and in 6.7% meiotic spindle was absent. In this group, there was a meaningful difference in comparison with the young control group (Figure 5). In group 6, of 60 retrieved oocytes, 87.1% were barrel-shaped, 11.7% showed decreased meiotic spindle size, 5% showed disruption in the meiotic spindle, and in 3.7% meiotic spindle was absent. In group 7, of 87 retrieved oocytes, 83.9% were barrel-shaped, 8% showed decrease meiotic spindle size, 4.6% showed disruption in the meiotic spindle, and in 3.4% meiotic spindle was absent. In group 8, of 96 retrieved oocytes, 79.2% were barrel-shaped, 9.4% showed decrease meiotic spindle size, 7.3% showed disruption in the meiotic spindle and in 4.2% meiotic spindle was absent. Statistical analysis showed that DHEA had a positive effect on barrel shape of the meiotic spindle. Also, we saw that aging process has a destructive effect on normal meiotic spindle shape and structure indeed (*P*=0.000). This effect decreased after administration of DHEA in old groups (*P*=0.000). There was not a meaningful difference in absence of meiotic spindle in young and old groups (Figure 6). The number of retrieved oocytes had a meaningful difference in young and old groups (*P*=0.009).

## Discussion

The present study evaluated the effect of DHEA on MII meiotic spindle structure of mouse oocytes. There has been an increase in reports about the benefits of DHEA in improving ovarian function among poor ovarian responders (14, 26). Barad and Gleicher described improved ovarian function among patients with DOR after DHEA administration (14). The action mechanism of DHEA on the ovary remains theoretical (27). Evidence shows that DHEA levels decrease with age (28). DHEA can improve steroidogenesis since it is a precursor of sexual hormones (29).

During ovarian induction with exogenous gonadotropins, DHEA is the prohormone of the follicular fluid testosterone (30). Androgens may influence ovarian follicular growth by serving as ligands for androgen receptors (31). Another possible mechanism was described by Casson *et al*, who described a temporary increase in insulin-like growth factor 1 (IGF-1) in patients undergoing exogenous gonadotrophin ovulation induction after pre-treatment with DHEA. This increase in IGF-1 may have been due to an increase in androgen production. They later hypothesized that the beneficial effect of DHEA may have been mediated by an increase in IGF-1 (8, 32). Goodarzi *et al.* claim that the effect of DHEA was due to the creation of polycystic ovarian syndrome (PCOS)-like characteristics in the aging ovary (33). Polycystic ovaries have an alteration at the transition from primordial to the primary follicle. Possible mechanisms that have been suggested for this observation are abnormal levels of growth factor, abnormally increased luteinizing hormone levels, or increased ovarian androgens (34). Ovarian theca cells of the pre-antral follicle produce androstenedione, testosterone, and DHEA**. **Higher levels of these androgens were found in the serum and ovarian veins of patients with the polycystic ovarian syndrome compared with controls.Long-term androgen exposure can induce histological and sonographic changes in normal ovaries similar to PCOS (35). The effect of DHEA is cumulative as more of the antral follicles become exposed to treatment, as described by Barad and Gleicher (36).

Reviewing the literature makes it clear that despite numerous studies of DHEA’s effects in a variety of human and animal studies, there remain many questions about its possible molecular mechanisms of action to be resolved (37). Studies show that DHEA blood serum levels decrease with age (38). This hormone, indeed, can improve steroidogenesis because it is a precursor of sexual hormones(estradiol and testosterone) (39). In our study, the oocyte retrieved rate improved after administration of DHEA in the old group. Our findings in this study were similar to achievements by Barad and Gleicher (5).

Huang *et al*. in 2015 found that a quite high dose rate of DHEA could interrupt mother metabolism and reduce pregnancy chances by induction of PCOS. They showed, however,that metformin treatment might reverse the impairment. Whereas, their study results confirmed that high dose DHEA-induced disturbance did not have a negative effect on meiotic spindle apparatus (40).

Many factors like cytoplasm and environment could affect the meiotic spindle.The previous studies showed that the aging process could have a negative effect on meiotic spindle and chromosomal alignment (23). In the presentstudy, we provide evidence that 10% of aged female mice (14 months old) have an abnormal meiotic spindle shape in comparison with control and other DHEA treated groups; these findings are like what was achieved in another study (41). Results, indeed, show that 90% of all meiotic spindles in both young and adult groups have a regular formation in microtubule and chromosomal alignment.We saw that there is a meaningful difference in meiotic spindle disruption between both control groups, these changes were eliminated after administration of DHEA in other studied groups, indeed, these findings prove our hypothesis about the positive effects of DHEA on the meiotic spindle in the aging process. We expected this hormone (DHEA) to have a positive effect on meiotic spindle structure, because the action mechanism of this hormone (DHEA) was unknown to previous researchers like Barad and Gleicher (5). But it is possible that other factors, such as mitochondrial and cytoplasmic factors could affect the meiotic spindle related aneuploidy. In this study, we saw a meaningful difference in meiotic spindle morphology between young (80%) and old (26.7%) control groups. This finding matches what was achieved in a study in 2010 about age-related effects of DHEA on peripheral markers of oxidative stress (42). Statistical analysis showed that there were no differences between the three given doses.The ability to revoke oocyte aging was first explored by researchers who found that oocyte aging can be reversed by controlling the activity of the maturation-promoting factor (43). Other investigators found that maturation-promoting factor and mitogen-activating protein kinase are essential for maintaining oocytes arrested at MII, as their activities gradually decreased during oocyte aging (44). Aging-related infertility is another cause of unsuccessful patient ART cycles (45).

At first, like other studies, we proposed that oocyte morphologic criteria are responsible for increasing low ART chance rates. Morphological evaluation before ART is useful for selecting MII oocytes with higher developmental potential (19, 46). Oocyte aging has been associated with several morphological and functional changes, including alteration in the structure of the plasma membrane, ZP, cytoskeleton, or mitochondria displacement of the first polar body and cortical granules (47). A reduced volume of intracellular compartments has been shown with aging in body composition studies (48). We also, indeed, assessed the DHEA serum levels in control, young, and old groups by the electrochemiluminescent method (data not shown in the results), then we found that serum levels of this hormone (DHEA) are different. These findings are similar to what was achieved by other researchers (9, 49).

## Conclusion

This study demonstrates that DHEA has a positive effect on meiotic spindle morphology and retrieved oocytes in old mice.
